# Impact of Abortion Bans on Emergency Care for Patients With Vaginal Bleeding: An Ethnographic Analysis

**DOI:** 10.1111/acem.70328

**Published:** 2026-06-05

**Authors:** Andreia B. Alexander, Kathryn J. LaRoche, Risa Cromer, Brownsyne Tucker Edmonds, Lori Freedman

**Affiliations:** ^1^ Department of Emergency Medicine Indiana University School of Medicine Indianapolis USA; ^2^ Department of Public Health Purdue University West Lafayette USA; ^3^ Department of Anthropology Purdue University West Lafayette USA; ^4^ Department of Obstetrics and Gynecology Indiana University School of Medicine Indianapolis USA; ^5^ Department of Obstetrics and Gynecology and Reproductive Sciences University of California San Francisco San Francisco California USA

## Abstract

**Background:**

The Dobbs decision enabled widespread state abortion bans, creating legal ambiguity for clinicians managing reproductive emergencies in the ED. While impacts on obstetricians are documented, less is known about how these laws influence emergency medicine practice. We explored how ED clinicians navigate care for patients with vaginal bleeding under Indiana's restrictive abortion ban.

**Methods:**

We conducted a team‐based ethnographic study at an urban academic ED and Level 1 Trauma Center in Indiana between August 2023 and October 2024. Data included six weeks of site‐based observations and semi‐structured interviews with 20 emergency clinicians. Interviews explored clinical decision‐making, documentation practices, and perceptions of legal risk following implementation of the abortion ban. Transcripts and field notes were analyzed using an iterative, consensus‐based qualitative coding process informed by ethnographic methods.

**Results:**

Three interrelated themes shaped clinician decision‐making: knowing, documentation, and reporting. Clinicians described altered approaches to history‐taking, particularly around self‐managed abortion, balancing medical relevance against concerns for patient stigma and legal harm. Documentation practices were characterized by uncertainty and fear, with many clinicians intentionally limiting chart detail to protect patients while simultaneously worrying about medico‐legal vulnerability. Knowledge of state‐mandated reporting requirements varied widely, contributing to anxiety, misinterpretation of the law, and defensive clinical behaviors. Across themes, clinicians reported persistent tension between ethical obligations to patient safety and autonomy and perceived pressure to ensure legal self‐protection. Robust institutional support was identified as a critical mitigating factor.

**Conclusions:**

Indiana's abortion ban has introduced legal uncertainty into emergency care for patients with vaginal bleeding, reshaping clinician behavior around information gathering, documentation, and reporting. These shifts risk delayed or defensive care, erosion of patient trust, and widening inequities in time‐sensitive emergency settings. Clear institutional guidance, interdisciplinary collaboration, and legal support are essential to safeguard evidence‐based emergency care and uphold ethical practice in restrictive reproductive policy environments.

## 1 Introduction

In June of 2022, the Supreme Court of the United States issued its decision in *Dobbs v Jackson Women's Health Organization*, overturning *Roe v. Wade*. This ruling allowed individual states to legislate abortion, and as of January 2026, 19 states have banned or significantly restricted abortion, with other legal cases pending in state courts across the country [1]. This evolving and ambiguous legal terrain has created uncertainty for clinicians providing reproductive care [2, 3, 4], particularly in emergency care settings [5, 6, 7, 8]. For clinicians in these settings, uncertainty goes beyond the delivery of abortion care itself to encompass the management of common reproductive emergencies, such as miscarriages, ectopic pregnancies, and pregnancy complications, that may clinically resemble or intersect with abortion care [9].

Emergency Departments (EDs) are uniquely impacted by the downstream effects of abortion restrictions. As the first point of contact for many patients experiencing pregnancy‐related complications, including miscarriage, ectopic pregnancy, and post‐abortion emergencies, EDs play an increasingly critical role in reproductive health care [9]. The closure of reproductive health centers and labor and delivery units across the United States, particularly in states with abortion bans [10, 11], may shift more pregnant patients to seek care in emergency settings. Policy‐level analyses show concurrent rises in overall ED utilization and obstetric‐related EMTALA complaints in states with restrictive abortion bans, suggesting a shift of acute pregnancy‐related care into emergency settings [12]. A growing volume of complex, time‐sensitive cases requires ED clinicians to make rapid, high‐stakes decisions while simultaneously navigating evolving legal and ethical uncertainties [13, 14].

Research examining the effects of abortion bans on clinical decision‐making demonstrates that such restrictions often compel clinicians to prioritize legal self‐protection over evidence‐based practice, leading to delays in care [15, 16, 17], constrained counseling, and compromised patient safety [3, 18, 19]. These laws have also curtailed clinicians' ability to provide comprehensive counseling and referrals for appropriate care, undermining patient autonomy and shared decision‐making [3, 19, 20, 21]. Beyond clinical implications, abortion bans have profound personal effects on clinicians, contributing to moral distress [22, 23], fear of violating ambiguous laws [3, 24], intentions to leave restrictive states, and symptoms of anxiety and depression [3]. However, while growing evidence has illuminated these effects among obstetric and gynecologic clinicians, little is known about how abortion bans influence the practice of emergency medicine. This gap is critical, as emergency clinicians frequently encounter pregnancy‐related emergencies under conditions of diagnostic uncertainty and time‐sensitive decision‐making.

Understanding how ED clinicians interpret and operationalize abortion‐related laws is essential to ensure patient safety, reduce inequities, and protect trust in emergency care. Additionally, qualitative inquiry can uncover nuanced ethical, emotional, and institutional dynamics shaping clinician behavior. Therefore, the aim of this study is to use ethnographic methods to explore how emergency clinicians understand and navigate care for patients presenting with vaginal bleeding in the context of Indiana's abortion ban—a total ban with exceptions for life and serious health risks of the pregnant patient, lethal fetal anomaly up to 20 weeks post‐fertilization (22 weeks by LMP), and rape or incest up to 10 weeks post‐fertilization (12 weeks by LMP) that went into effect on August 21,2023 [25].

## 2 Methods

### 2.1 Study Design

We conducted a team‐based ethnographic study [26, 27] at a hospital within a major academic medical center in Indiana from August 2023 through October 2024. We drew on observations and interviews to understand how a new abortion law impacted practice. The team included an emergency medicine physician (AA), an ob‐gyn physician (BTE), a sociologist (LF), a public health researcher (KL), and an anthropologist (RC). The study was approved by the Institutional Review Board of Indiana University. No patient identifiers were collected for this study.

### 2.2 Study Setting and Population

Our hospital site was an academic ED at a Level 1 Trauma Center and Tertiary Care Center in an urban area. Pregnant patients commonly present to this ED with vaginal bleeding concerns. We observed clinical flow and clinician‐patient interactions when possible (excluding during physical exams). During observations, we recruited clinicians for a later confidential telephone interview according to a purposive sampling strategy aimed at acquiring a well‐rounded set of perspectives, balanced for both demographics and clinician types.

Using a semi‐structured interview, LF interviewed emergency medicine clinicians, including registered nurses, advanced practice providers, residents, attending EM physicians, and ob‐gyn hospitalists who are regularly consulted in the hospital's ED. The interview guide focused on (1) approaches to caring for vaginal bleeding patients; (2) descriptions of cases that were impacted by the abortion law change (if at all); (3) interviewees' professional views on and concerns about the law change and impact on patient care; and (4) strategies clinicians used to ensure patient safety and wellbeing in this new medicolegal context. The team worked together to write interview instruments and iteratively revise them at a few points during the interview process, deleting less useful questions and adding missing ones. Interviews were conversational, allowing for clarification of responses in real time and the discovery of unforeseen areas of explanation. The original interview guide can be found in Data S1.

Once clinicians agreed to be contacted for an interview, LF reached out to schedule it. Interviews took place over the phone and lasted approximately 1 h. Clinicians were given a $50 gift card for their participation. Interviews were audio recorded with the participant's permission and then transcribed by a contracted (non‐AI) service. We reviewed the transcripts for accuracy and reacted all information that could be used to identify people or institutions.

### 2.3 Data Analysis

The team worked together to generate a priori codes through discussion of the interviews. LF, RC, and KL reviewed three transcripts line‐by‐line to create a provisional code book, and they applied codes to an additional transcript. They went through this process six times, reviewing each other's coding style and settling disagreements through consensus. The coders ultimately settled on the sixth version as the final codebook and divided up the transcripts between them to complete the coding in a qualitative data management software program. The researchers met weekly to discuss any questions and memos created during the process that required deliberation to reach consensus. For this manuscript, AA and LF analyzed output labeled *know*, *document*, and *reporting* for this manuscript.

## 3 Results

Throughout this section, we use ‘vaginal bleeding’ to refer to the full spectrum of presentations, whereas ‘self‐managed abortion (SMA)’ denotes a specific subset of cases discussed by clinicians within this broader category.

We analyzed 20 ED clinician interviews and six weeks of site visit field notes (41 single‐spaced pages) taken over 14 months for this manuscript. Participant demographics are found in Table 1. Overall, we identified multiple themes in the data; however, this manuscript will focus on three key findings relevant to provider decision‐making regarding the care of vaginal bleeding patients in the ED: know, documentation, and reporting.

**TABLE 1:** Demographics.

Characteristic	Overall *N* = 20
Age, *range (mean)*	
	24‐64 (42)
Sex, *n (%)*	
Female	15 (75)
Male	5 (25)
Non‐Binary	0 (0)
Race, *n (%)*	
Black	3 (15)
Non‐Hispanic white	17 (85)
Profession, *n (%)*	
ED Advanced Practice Provider	4 (20)
EM Attending	10 (50)
OB Hospitalist	2 (10)
ED Registered Nurse	2 (10)
EM Resident	2 (10)
Years in practice, *n*	
Less than 2 years	2 (10)
2 to 5 years	2 (10)
6 to 10 years	5 (25)
More than 10 years	11 (55)

### 3.1 Know

The code *Know* captured how clinicians described obtaining and interpreting relevant patient information, including the types of information sought, associated concerns, and approaches to patient communication. Across interviews, clinicians consistently described seeking information typically used to assess vaginal bleeding: initiation, description, pads per hour, signs/symptoms of anemia, pregnancy status, pregnancy history, and prior miscarriages.

When discussing suspected self‐managed abortion (SMA), many clinicians reported that determining whether a patient had self‐managed was not necessary for clinical care. Clinicians indicated that they would incorporate such information if voluntarily disclosed but generally did not directly ask about SMA, as management of vaginal bleeding would remain the same regardless of etiology. As one clinician explained:

“…a patient with vaginal bleeding, it's vaginal bleeding. It doesn't really matter how we got here. We're going to treat it the same.” ED11

A few clinicians described selectively exploring whether patients had taken medications if this information was clinically relevant to immediate care. This was most often discussed in the context of potential exposure to harmful substances (e.g., pennyroyal) or trauma, which could require additional evaluation or intervention.

Clinicians also described potential harms associated with directly eliciting information about SMA. These harms were described in two primary ways.

First, clinicians expressed concern about the emotional impact of such questioning on patients experiencing miscarriage. Specifically, they noted that asking about intentional pregnancy termination could inadvertently reinforce feelings of self‐blame among patients with desired pregnancies. One clinician described this tension:

“I don't have the dialogue yet in my mind of how to gently ask someone, ‘Was this an intentional outcome of something you did?’ without accidentally telling some other woman with a very desired pregnancy inferring that they may have caused the loss of their pregnancy.” ED05

Second, clinicians described concerns about potential legal risks to patients who disclosed self‐managed abortion. As a result, many clinicians reported avoiding direct questioning and instead attending to information patients voluntarily shared, while attempting to create a clinical environment that allowed patients to disclose information if they chose to do so.

A minority of clinicians emphasized the importance of obtaining as much information as possible to ensure clinical accuracy. As one participant noted:

“I would err on the side of give me all the details. You know, we are going to deal with the medical side of this now and figure out this whole reporting thing later.” ED07

### 3.2 Documentation

The code *Documentation* captured how clinicians described what they did or did not include in the patient medical record. In discussions of documentation, nearly all ED clinicians expressed uncertainty regarding what information they were required to document for patients presenting with vaginal bleeding. This uncertainty was most pronounced in relation to documenting discussions of self‐managed abortion (SMA) and travel out of state for abortion care. As one clinician described:

Respondent There was a case recently with a patient who was a bit further along in pregnancy and interested in going out of state for termination. And I think what kind of struck me about that case was there was a lot of anxiety about what to document in the chart, what to include, what not to include, those sorts of things.

Interviewer What was considered concerning to people?

Respondent Everybody was just very worried about inadvertently, I think, putting something in the charts that could endanger the patient, essentially

Interviewer Worried about her protection?

Respondent Yes. That by even just mentioning that this was not a wanted pregnancy that she could get in trouble. ED08

Clinicians described varied interpretations of Indiana's abortion laws, which contributed to uncertainty regarding documentation practices. While a small number of clinicians expressed confidence that the law criminalizes providers rather than patients, others reported uncertainty or misinterpretation. For example, some clinicians (incorrectly) thought it was illegal for the patient to self‐manage an abortion in the state of Indiana, as one clinician stated:

“I don't know. Is charting that they got an abortion incriminating her? Probably. But I don't know what the laws are going to look like with that. How are they going to have access to patients' medical records? Is it only going to be if they've heard that somebody got an abortion, that they're going through these records?” ED01

Many thought the language of the laws was in place to scare providers.

Many clinicians described tension between documenting sufficient clinical detail and protecting patient safety. This tension was often framed as a need to balance accurate medical documentation with concern about potential downstream legal consequences. One clinician explained:

“I know I personally do not ask patients anymore if they had an abortion or if they had taken any medications or anything like that. Because I do not want to be legally responsible for reporting them to the state, because I don't feel like that's my place.” ED14

The same clinician further described:

“We had a woman who had very, very significant medical conditions that made it [so] she would have died if she had continued the pregnancy. I …made sure that I certified in [the patient's chart] that this is a life‐threatening condition in multiple ways. And [I] explained it in multiple ways. So that if somebody did come back and [take] a look at her chart after she had the termination, that the patient would be okay. I don't want [the patient] to ever have somebody come back and punish her for trying to save her own life.” ED14

Clinicians were concerned about the legal ramifications of both under‐and over‐documentation. A subset of clinicians reported modifying their documentation practices in response to the legal environment. These changes included limiting documentation of certain contextual details, such as whether a pregnancy was desired. This often led to reduced documentation of the social and emotional context of the care, as exemplified by this clinician:

“Honestly, I wouldn't document much about it before [the ban]. I certainly document absolutely nothing about it now. At this point it is strictly,” “They are pregnant. It is a viable intrauterine pregnancy at X weeks. They were given prenatal vitamins and referred for outpatient counseling or follow‐up.” ED03

To expand on this point, the same clinician said:

“My primary obligation is to the patient and to the hospital system to accurately document the medical necessity—like, the medical facts….that's the only thing they use for billing and coding. The only thing that they use is my decision‐making”. ED03

Across interviews, clinicians consistently described efforts to ensure that documentation practices did not compromise patient safety or trust, even as they navigated uncertainty about legal expectations.

### 3.3 Reporting

Indiana requires that clinicians submit a Termination of Pregnancy Report (TPR).^25^if they perform an abortion, and report any patient complications on the Abortion Complication Report (ACR). The code *Reporting* captured clinicians' descriptions of their understanding of state‐mandated reporting requirements, including TPRs and ACRs, as well as perceptions of when and how reporting should occur. This code also included clinician perspectives on reporting patients to law enforcement.

Clinicians described substantial variability in their understanding of reporting requirements. Reported knowledge ranged from lack of awareness of reporting processes to misinterpretation, partial understanding, and reliance on consultants, particularly OB/GYN physicians, to manage reporting responsibilities.

For example, this clinician showed an accurate (though not confident) interpretation of the laws by explaining:

“There is, to my knowledge, no mandatory reporting for miscarriages. So, if they are sick enough to need a gynecologist, which would probably be the sort of thing that would mandate reporting, then they need a gynecologist. And if they're not sick enough to need a gynecologist, the odds that they need any sort of reporting are relatively small, because they're not sick.” ED03

Other clinicians described observing or experiencing confusion among colleagues regarding legal requirements, including uncertainty about whether discussing or recommending abortion‐related care was permissible:

“The way that people misunderstand the misinformation and disinformation around all of the laws in the state are insane. I had a provider the other day who…was like, “Is it even legal for me to recommend she has an abortion?” for this patient [who] was incredibly sick. She's like, “I have little kids. I don't want to go to jail.” It's like they completely extrapolate…So, all these things that are not even in the law, there's nowhere in the law that says you can't recommend, offer resources, et cetera, but people just freak out.” ED09

Clinicians also described reporting requirements as cumbersome to complete and meant to trick them. While some participants compared abortion‐related reporting to other forms of mandatory reporting (e.g., dog bites, measles), most emphasized that abortion‐related reporting carried different perceived stakes including fear of prosecution, harassment on websites, and potential effects on their families, as expressed by this clinician:

“But if I make an error in reporting a dog bite, I'm not worried that would be the end of the world for me. I'm sure someone would point that error out to me, and I would correct that going forward in the future. But [abortion] it's such a charged issue. I will be honest: when you have questions surrounding the reporting of some potential complication from an elective abortion procedure, it **is** scary as a physician. If I don't do this right, am I going to be on the news? Is the attorney general going to be knocking on my door? It is a higher stakes thing, and I don't like that.” ED16

As a result, many ED clinicians discussed relying on the OB/GYN consultants to complete these reporting requirements.

In addition to mandated reporting processes, clinicians described uncertainty regarding whether and when patients should be reported to authorities for self‐managed abortion. Some clinicians believed that they might be required to report patients if self‐management was suspected, while others described uncertainty about their legal obligations in these situations. One clinician described this uncertainty:

“I've had the panics of, ‘Am I going to get in legal trouble because I have missed something? Because I had someone come in who's got vaginal bleeding who's pregnant and maybe they did try to terminate this pregnancy and I didn't catch it? But am I supposed to report all pregnant women with vaginal bleeding to the government?…Am I supposed to know when they're like, by the way, I bought this medication online? Or, by the way, I drove across state lines and came back? If I don't ask that information, they're not going to offer it up. But if I do know that information, legally I have to do something about it.” ED05

Other clinicians described understanding (correctly) that self‐managed abortion is not a criminal offense in Indiana and emphasized efforts to reassure patients in these situations.

Across interviews, clinicians frequently described misalignment between legal reporting requirements and clinical care priorities, as stated by this clinician:

“What we think medically isn't always the same as what some people at the state level think legally.” ED20

Reporting requirements, in general, made many clinicians uncomfortable and affected the way they cared for the patient. Regardless, protecting patient trust appeared to be at the top of mind for many clinicians, as expressed by this clinician:

“I know, for me personally, because I do not want to ever destroy the trust that a woman has in the medical system, I would probably err on the side of being a little bit more vague with my answers to some of those questions to make sure that she was being protected because I know, theoretically, they tell you that they can't, you know, identify women individually or, you know, do any of that stuff from these registries that they're creating, but there's always that in the back of your head.” ED14

### 3.4 Overall Effects of Abortion Laws on ED Clinicians

**FIGURE 1:**
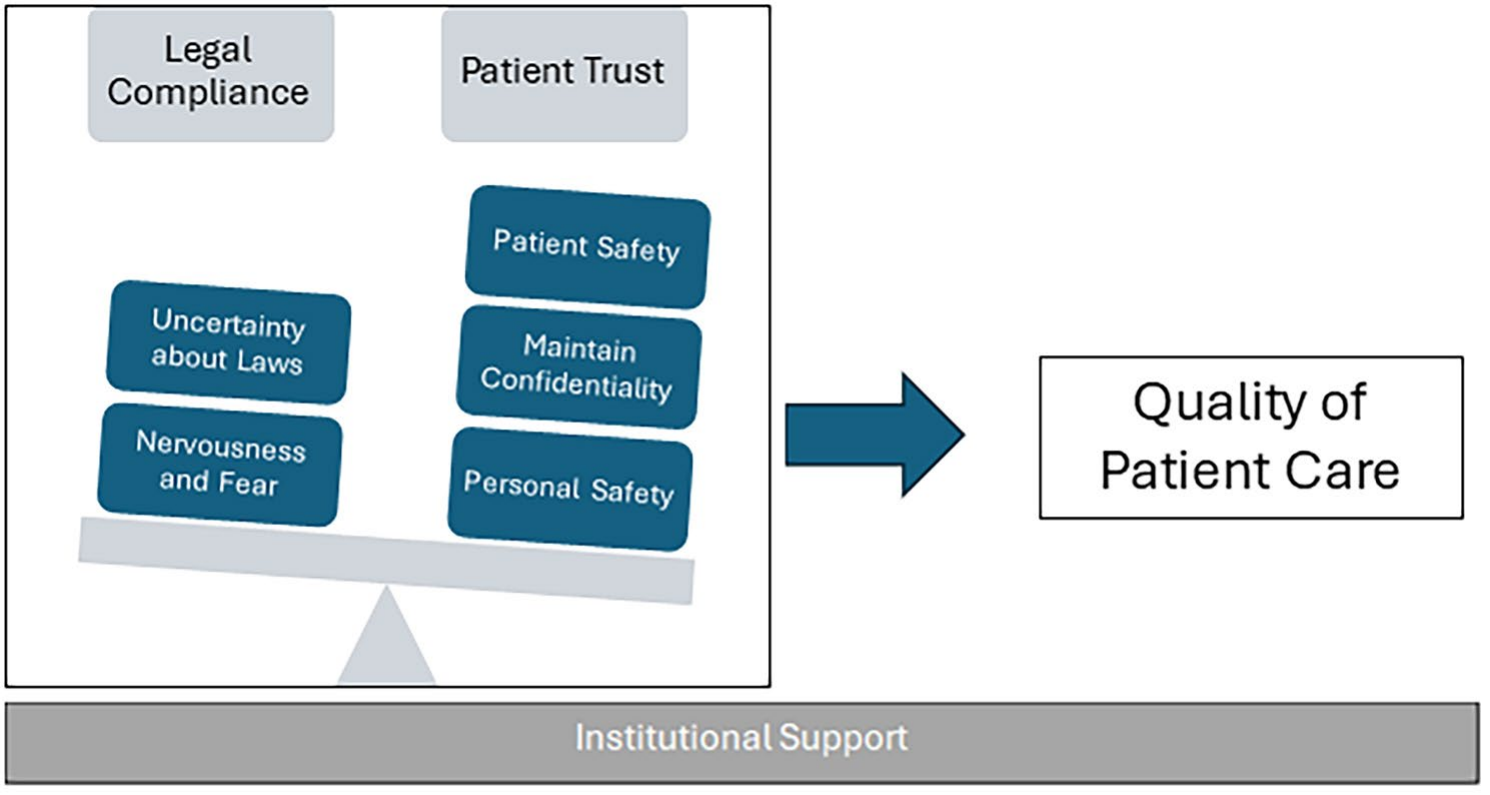
Conceptual model of how abortion‐related legal uncertainty shapes ED clinician decision‐making. This model synthesizes findings from clinician interviews, demonstrating how the domains of knowing, documentation, and reporting are influenced by a balance between perceived legal risk and maintaining patient trust. These factors interact to shape clinical behaviors. These processes contribute to changes in care practices, including altered information gathering, modified documentation, and variability in reporting, with implications for patient trust and safety.

Across the three codes—knowledge, documentation, and reporting—clinicians described the ongoing challenge of reconciling legal compliance with the ethical imperatives of patient care, trust, and safety. This tension was often mitigated by robust institutional support structures (Figure 1). Overall, clinicians demonstrated substantial uncertainty regarding the scope and application of Indiana's abortion laws, which contributed to pervasive anxiety about potential legal, professional, and personal repercussions for both themselves and their patients.

Clinicians articulated a strong commitment to maintaining patient trust, emphasizing confidentiality and patient safety as essential to ethical practice. However, this commitment frequently intersected with concerns about self‐protection under the law, producing strong internal conflict. Decisions around documentation were particularly fraught: clinicians grappled with how much detail to include about a patient's presentation or reproductive history. They reasoned that less‐detailed documentation could undermine their ability to defend clinical decisions in potential legal proceedings, yet excessive documentation risked compromising patient confidentiality and safety.

Competing obligations to protect patients and themselves often manifested in altered clinical practice patterns. Several clinicians reported delaying care or pursuing defensive strategies, such as consulting obstetrics colleagues unnecessarily because, “I just don't want to deal with it,” or defaulting to expectant management for miscarriage even when medication or surgical management may have been indicated or preferred. Despite these challenges, nearly all participants identified strong institutional support—particularly from accessible legal counsel and a responsive OB/GYN team—as a critical buffer against the emotional and professional strains of navigating care provision under restrictive abortion laws.

## 4 Discussion

This study highlights that abortion bans create immediate, tangible impacts on the delivery of emergency care. As the primary point of entry for patients experiencing early pregnancy complications, EDs are uniquely sensitive to the chilling effects of restrictive reproductive laws [14]. Our findings reveal a professional landscape defined by tension as clinicians are caught between navigating vague, potentially punitive legal mandates and fulfilling their ethical commitment to patient safety and autonomy.

Clinicians in our cohort exhibited behavioral shifts, including delaying care, pursuing defensive strategies, and increasing unnecessary consultations to protect themselves from legal repercussions. These behaviors align with established behavioral research on decision‐making under constraints, which suggests that when faced with high‐stakes ambiguity, individuals often succumb to motivated reasoning, risk aversion, and decision paralysis [24]. By shifting the focus from evidence‐based care to legal self‐protection, these constraints compromise patient agency and choice, and potentially patient safety. This shift is particularly dangerous in emergency settings where lives and morbidity are at risk. Our data suggest that the fear of the law functions as an extra‐clinical factor that can lead to sub‐optimal health outcomes, echoing findings that abortion restrictions compel clinicians to prioritize legal safety over established medical standards [12]. This phenomenon will be more fully explored in a future manuscript by this team.

Our key findings labeled “*Know”* reveal a significant dilemma regarding information solicitation. While most clinicians expressed a desire to understand the medical history of a SMA to ensure patient safety (e.g., identifying exposure to harmful substances like pennyroyal), they simultaneously recognized that probing for this information could compound distress in patients who are having a miscarriage. Seeking information about SMA can trigger profound negative emotions and reinforce the stigma of self‐blame in patients experiencing pregnancy loss [28]. Furthermore, as documented in previous work [29], potential and actual legal risk shifts the intent of information‐gathering from a care‐based inquiry to a protection‐based inquiry. When the intent of the inquiry shifts toward protection, the patient‐physician relationship is often damaged [30]. As seen in situations outside of reproductive care, such as suspected child abuse or domestic violence, this phenomenon, referred to as the “forensic turn,” describes the moment a clinical encounter becomes an evidentiary one. Studies have shown that the shift to an investigatory mindset fundamentally alters the clinician's questioning style [31, 32], often resulting in patients withholding information, further fracturing the patient‐physician relationship [33, 34]. Patients in Indiana do not currently face legal ramifications for obtaining a SMA. However, ensuring that clinical encounters remain grounded in care rather than compliance will be essential to preserving both ethical practice and high‐quality reproductive health care should the legal landscape surrounding SMA shift.

Decision‐making regarding *documentation* was particularly fraught with anxiety. Our findings indicate that clinicians grapple with a “double‐edged sword”: insufficient documentation might fail to defend a clinical decision in court, while excessive documentation of a non‐viable or terminated pregnancy could inadvertently create a forensic trail that endangers the patient or exposes the physician to prosecution for “aiding and abetting” a termination [12, 29, 35]. This uncertainty stems from the unclear and evolving language of the law, which lacks clinical precision, leading to varied and often conflicting interpretations among ED staff [5, 36]. The variability in how clinicians interpret or misinterpret (e.g., SMA) the law and what must be *reported* creates the potential for unequal care and unnecessary legal exposure for patients [37]. Although SMA is not currently illegal in Indiana, and reporting patients to law enforcement is a HIPAA violation, the evolving landscape and legal precedence in neighboring states for prosecuting abortion‐seekers creates a near and present perception of risk and danger. This is exacerbated by the highly politicized environment in which clinicians fear harassment, professional ruin, or prosecution by state authorities [38].

Despite evident fear, *institutional support* emerged as a critical buffer. In contrast to literature suggesting that institutional guidance is often variable or vague [39, 40], our participants identified robust support, specifically from legal counsel and OB/GYN teams [41], as essential to mitigating moral distress. Strengthening collaborations between ED clinicians, OB/GYN specialists, and hospital administration can reduce the cognitive load on individual physicians and promote safer, more consistent clinical practices. Clear internal policies and just‐in‐time training are vital to supporting clinicians as they navigate this new and evolving medico‐legal reality.

## 5 Limitations

Although this study provides an in‐depth qualitative look at the intersection of emergency medicine and restrictive abortion laws, several limitations must be considered. This study was conducted within a single major academic medical center in Indiana. Consequently, the findings regarding clinician uncertainty and institutional support may not be generalizable to rural hospitals, community emergency departments, or facilities in states with different legal frameworks. We used a purposive sampling strategy to recruit clinicians. It is possible that clinicians with stronger views on abortion laws or those experiencing higher levels of moral distress were more likely to participate, potentially skewing the breadth of perspectives. Because the legal environment is described by participants as “scary” and high‐stakes, clinicians may have been hesitant to fully disclose practices that could be perceived as legally ambiguous, despite assurances of confidentiality (social desirability bias). Furthermore, interview data relied on participants' retrospective accounts of their clinical encounters and decision‐making processes (recall bias). Finally, this analysis focused specifically on the key findings related to codes *Know, Documentation*, and *Reporting*. While these were identified as the most salient factors regarding physician decision‐making for vaginal bleeding care, other identified themes were not explored in depth here and will require future reporting.

## 6 Conclusion

Indiana's abortion ban has introduced legal uncertainty into emergency care for patients with vaginal bleeding, shifting clinicians' decision‐making around history‐taking, documentation, and reporting away from clinical priorities and toward legal protection. In the ED, where care is time‐sensitive and diagnostic uncertainty is common, these pressures risk delaying treatment, eroding patient trust, and exacerbating inequities. Clear institutional guidance and legal support are essential to ensure that emergency clinicians can provide timely, evidence‐based care without fear of legal repercussions.

The authors used IU Google Gemini for grammatical and stylistic revisions. All intellectual content, data analysis, and conceptual frameworks presented in this work are the original ideas of the authors, who maintain full responsibility for the integrity of the final manuscript.

## Author Contributions

A.B.A.: study concept and design, analysis and interpretation of the data, drafting of the manuscript, critical revision of the manuscript for important intellectual content. K.J.L.: study concept and design, acquisition of the data, analysis and interpretation of the data, critical revision of the manuscript for important intellectual content. R.C.: study concept and design, acquisition of the data, analysis and interpretation of the data, critical revision of the manuscript for important intellectual content. B.T.E.: study concept and design, critical revision of the manuscript for important intellectual content. L.F.: study concept and design, acquisition of the data, analysis and interpretation of the data, critical revision of the manuscript for important intellectual content, and acquisition of funding.

## Funding

This work was supported by Greenwall Foundation. Clifford B. Kinley Trust at Purdue University.

## Disclosure

This work was presented at the 2025 Society of Academic Emergency Medicine Annual Meeting in Philadelphia, PA.

## Conflicts of Interest

The authors declare no conflicts of interest.

## Supporting information


**Data S1:** Supporting Information.

## Data Availability

The data that support the findings of this study are available on request from the corresponding author. The data are not publicly available due to privacy or ethical restrictions.
