# Supplementary material for: Impact of Abortion Bans on Emergency Care for Patients With Vaginal Bleeding: An Ethnographic Analysis
Source: Acad Emerg Med. 2026 Jun 5;33(6):e70328. doi: 10.1111/acem.70328 (PMC13238399; doi:10.1111/acem.70328)
Supplement: Supplementary file 1 — Data S1: Supporting Information. [file ACEM-33-0-s001.docx]

Indiana Emergency Department and Labor & Delivery Provider Interview Instrument

Go through formal consent process. Then, state:

A. “Just to reiterate: this is meant to be more of a conversation about you and your work experiences than a survey. I’m interested in your perspective. There are no right or wrong answers. The interview is entirely confidential, and I will ensure that your responses are not identifiable. Your coworkers won’t know that you were interviewed unless you tell them. And any publications from this research will not identify the city or hospital by name. I plan to interview about 40 people. Do you have any questions for me before I start to record?”

1. To get started, so I can know a little more about you, could you tell me how you decided to become a nurse/doc/other healthcare worker? What was your professional path?
2. And how did you end up working in this Emergency Department or Labor & Delivery service? How long have you been working here?
3. How has your job changed, if at all, over that time?
4. Did you grow up around here? If not, how did you end up living in or near Indianapolis?
5. How does working here compare with the last place you worked? What do like better? What do you like less about it?

B. Vaginal bleeding patients

I want to ask you some questions about younger women who present with vaginal bleeding and need care for any reason? (prompt: could be early pregnancy loss, incomplete abortion).

1. CASE ONE: can you tell me about the most recent patient you remember who came in with like this? *(prompts, if not offered….)*
   1. What was going on with her physically?
   2. How was she behaving?
   3. What did she say about what had happened?
   4. What were your concerns in seeing her?
   5. Can you walk me through the course of her care while she was there?
   6. What do you wish you would have asked the patient that you didn’t? Why didn’t you?
   7. [continue to solicit as much detail as possible about the course of treatment, the interviewee’s thoughts about what happened, if abortion-related how that was discerned?]
   8. *If abortion related:* What procedures are typical for an abortion patient? Typically, do you have to tell anyone or report anything in case like this?
   9. How would you do anything differently, ideally, in your opinion in a case like this?
2. CASE TWO: Can you tell me about another case when a patient presented with vaginal bleeding or pregnancy complications that is particularly memorable? [solicit all same detail]
3. CASE THREE *(If not yet volunteered):* Can you recall a patient coming in for abortion complications or follow up care? What happened…. *[solicit all same detail a-i]*
   1. Did staff respond differently to this patient than other patients? How so?
4. CASE FOUR *(If not yet volunteered):* Can you recall a patient coming in who said she was miscarrying, but it seemed like she may have taken pills to induce an abortion? What do you remember about that case *[solicit all same detail a-i].*
   1. What behaviors would make you think that she’d induced an abortion?
   2. What about the patient makes you think it might be an abortion?
   3. What patterns have you identified in these patients?
5. When someone needs care for abortion complications, do you typically need to report that anywhere?
   1. What do you do?
   2. Who gets involved or needs to know?
   3. What paperwork do you do?
   4. What do you think happens to that report? Is there follow up?
   5. What happens to the patient?
   6. How do you feel about reporting abortion complications? Why?
   7. What would you change about that process? Why?
6. How has the documentation of patient care changed since abortion was made illegal in Indiana?
7. (If not addressed): How is this different than before it was illegal?
8. How do you feel about it?
9. How has the way you care for patients who may have had an abortion changed since it became illegal?
10. What are you concerns about the law for health care providers?
11. Have you noticed a difference in how such patients present for care since then? (stories, stages, things unspoken?)
12. How do the different types of health care providers you work with respond differently to abortion-related cases? (nurses, doctors, medical assistants or techs, social workers…)
13. We understand the patient population have various language needs, how has that impacted care for vaginal bleeding in ways that you’ve seen?
14. Do any abortions take place for any reasons in this hospital that you are aware of?
    1. Can you recall a case in your hospital?
    2. Please tell me about it? *[solicit all detail]*
    3. Are there any others you can think of? *[solicit all detail]*
15. Generally speaking, has patient care in your hospital changed since the abortion law changed?

***Returning to vaginal bleeding patients in general:***

1. How do you start the conversation with a patient who hasn’t offered much information yet about their vaginal bleeding?
   1. What do you want to know?
   2. What do you not want to know? Why?
2. Can you remember a time that you felt like an interaction with a VB patient was challenging?
   1. What made it challenging?
   2. Is it something that you’ve noticed in similar scenarios?
   3. What are some other things you’ve noticed that have been interpersonally challenging rather than clinically challenging?

18. What challenges have you noticed about patients receiving fair and equitable treatment in these emergency vaginal bleeding cases? (Why do you think this is an issues?)

19. Have you ever observed care for a vaginal bleeding patient and felt concerned a patient wasn’t treated fairly for some reason? If yes, do mind recounted how that went, without names of course? (Solicit details…)
